# Anatomic Flat Double-Bundle Medial Collateral Ligament Reconstruction

**DOI:** 10.1016/j.eats.2023.03.017

**Published:** 2023-07-03

**Authors:** Janina Kaarre, Armin Runer, Neilen A. Benvegnu, Sahil Dadoo, Fabian Persson, Michael Nammour, Volker Musahl, Kristian Samuelsson

**Affiliations:** a Department of Orthopaedics, Institute of Clinical Sciences, Sahlgrenska Academy, University of Gothenburg, Gothenburg, Sweden; b Sahlgrenska Sports Medicine Center, Gothenburg, Sweden; c Department of Orthopaedic Surgery, UPMC Freddie Fu Sports Medicine Center, University of Pittsburgh, Pittsburgh, Pennsylvania, U.S.A.; d Department of Sports Orthopaedics, Klinikum rechts der Isar, Technical University of Munich, Munich, Germany; e Department of Orthopaedics, Sahlgrenska University Hospital, Mölndal, Sweden

## Abstract

Several surgical techniques have been described to restore the anatomy of the medial collateral ligament, involving suture repair and reconstruction, with the latter having been associated with superior postoperative outcomes. Recently, a growing interest in anatomic isometric medial collateral ligament reconstruction (MCLR) has been developed, involving careful evaluation and finding the most appropriate location for the femoral placement of the allograft. Therefore, the purpose of this article is to describe anatomic MCLR aiming to restore medial knee stability by focusing on isometric positions within the native anatomy of the MCL.

## Technique Video

Video 1 Anatomic technique for medial collateral ligament (MCL) reconstruction performed using flattened double-bundle allograft such as peroneus longus or semitendinosus graft. Intraoperative physical examination includes valgus stress testing at 0° and 20°. The surgical procedure begins with the patient positioned supine on the operating room table. A skin incision is made extending from the medial femoral condyle to the pes anserinus. The fascia overlying the pes anserinus is thereafter opened in such a manner that it can be closed afterward. The anterior and posterior borders of the distal MCL are visualized, and a K-wire is placed at the center of the distal tibial insertion of the superficial MCL (sMCL), approximately 6 cm below the joint line. The femoral MCL insertion is visualized and palpated, and a K-wire is placed in the anatomic center of the MCL origin. Graft isometry is checked throughout joint range of motion by wrapping a suture around both K-wires. The ends of the suture are then clamped at the proximal femoral K-wire. Suture displacement should not exceed 1 to 2 mm as the knee is ranged from flexion to extension, starting from 30°. Once graft isometry has been confirmed, one 5.5-mm Healix Advance PEEK anchor and one 5.5-mm Healix Advance BR anchor with 4 nonabsorbable sutures and needles are placed: one at the distal tibial insertion of the sMCL and the other about 10 mm below the joint line, central to the course of the native MCL, at the proximal insertion of the sMCL. The femoral guidewire is removed and changed to a Beath pin to pass the sutures. The looped MCL allograft is thereafter placed and tied firmly to the distal tibial sMCL suture anchor. Each graft arm is additionally tied to the proximal tibial suture anchor to provide a second tibial attachment closer to the joint line. Both proximal graft ends are now whipstitched together at a length of at least 25 mm using a No. 2 nonabsorbable suture. Any excess graft should be removed at this point. The femoral guidewire is then over-reamed using a drill based on the diameter of the whipstitched graft. The lead sutures are shuttled through the bone tunnel and lateral soft tissue using the Beath pin, allowing the insertion of the graft into the bone tunnel. With the knee in 30° of knee flexion and neutral rotation, a fully threaded cannulated Bioresorbable Matryx Interference Screw is inserted over a nitinol guidewire. The sutures of the proximal and distal tibial suture anchors are now used to tie the MCL graft with whipstitches to the native MCL, followed by cutting of the remaining sutures, to obtain the final flat double-bundle MCL reconstruction.

The medial collateral ligament (MCL) is one of the most frequently injured ligaments of the knee,^1^ requiring careful evaluation and appropriate management. The MCL consists of 3 structures: superficial MCL (sMCL), deep MCL (dMCL), and posterior oblique ligament (POL).^2^ The MCL, originating on the medial epicondyle of the femur and inserting on the medial condyle of the tibia, is the most important static stabilizer of the medial site of the knee.^3^ Biomechanical studies have shown that the sMCL functions as a primary restraint to valgus stress^3^ whereas the dMCL contributes to controlling external rotation.^4^ Moreover, the POL has been shown to function as a secondary restraint to valgus and both internal rotation and external rotation together with the posterior medial capsule. Successful outcomes, such as improved knee function and knee-related quality of life, have previously been reported after both nonoperative treatment and operative treatment.^5^^,^^6^ However, MCL deficiency has been recognized to be one of the main contributors to an increased risk of failure after anterior cruciate ligament reconstruction (ACLR)^4^^,^^7^ owing to increased valgus instability of the knee and subsequent graft tension of the new graft,^4^ such as quadriceps and hamstring tendon autograft. Additionally, recent research has illustrated an increased risk of failure after revision ACLR in the setting of concomitant medial knee instability.^8^

Various surgical techniques have been described to restore the anatomy of the MCL, such as suture repair and reconstruction, with the latter having been associated with superior outcomes.^9^^,^^10^ Thus, reconstructing the injured MCL and re-creating the normal anatomy of the sMCL have been recognized to be essential to avoid additional injuries and failures.^11^ Furthermore, MCL reconstruction (MCLR) performed with flattened broad graft has previously been associated with superior outcomes, including better control of valgus stability and external rotation, compared with single-bundle MCLR.^2^ This is a result of the greater amount of collagen making the graft thicker and more durable. Therefore, the aim of this article is to present an anatomic technique for MCLR, developed by the senior author (K.S.), performed using flattened double-bundle allograft such as peroneus longus or semitendinosus graft.

## Surgical Technique

### Positioning and Examination

The patient is positioned supine on the operating room table with the ability to flex and extend the knee between 0° and 100°. After induction of general anesthesia, a well-padded tourniquet is placed around the thigh and knee examination is performed including ligament tests, such as the valgus stress test at 0° and 20°. The lower extremity is thereafter prepared and draped according to standard sterile routine.

### Graft Choice

Possible graft choices include both autograft and allograft. Autograft options include the semitendinosus tendon and the anterior half of the peroneus longus tendon. We, however, prefer allograft to reduce donor-site morbidity and to prevent weakness on the medial side of the knee. The semitendinosus tendon is an important dynamic stabilizer to valgus stress^12^ and is preferably protected after an MCL injury. The preferred allograft option is peroneus longus allograft because of its greater thickness. The graft should be at least 25 cm in length, depending on the size of the knee.

### Graft Preparation

The allograft is opened and thawed in normal saline solution. The graft is placed on a graft preparation station and measured to the appropriate length of at least 25 cm. If an autograft is used, all muscle tissue should be removed. The graft is looped, providing an about 9- to 10-mm-thick, double-bundle construct.

### Tunnel Placement

**Fig 1:**
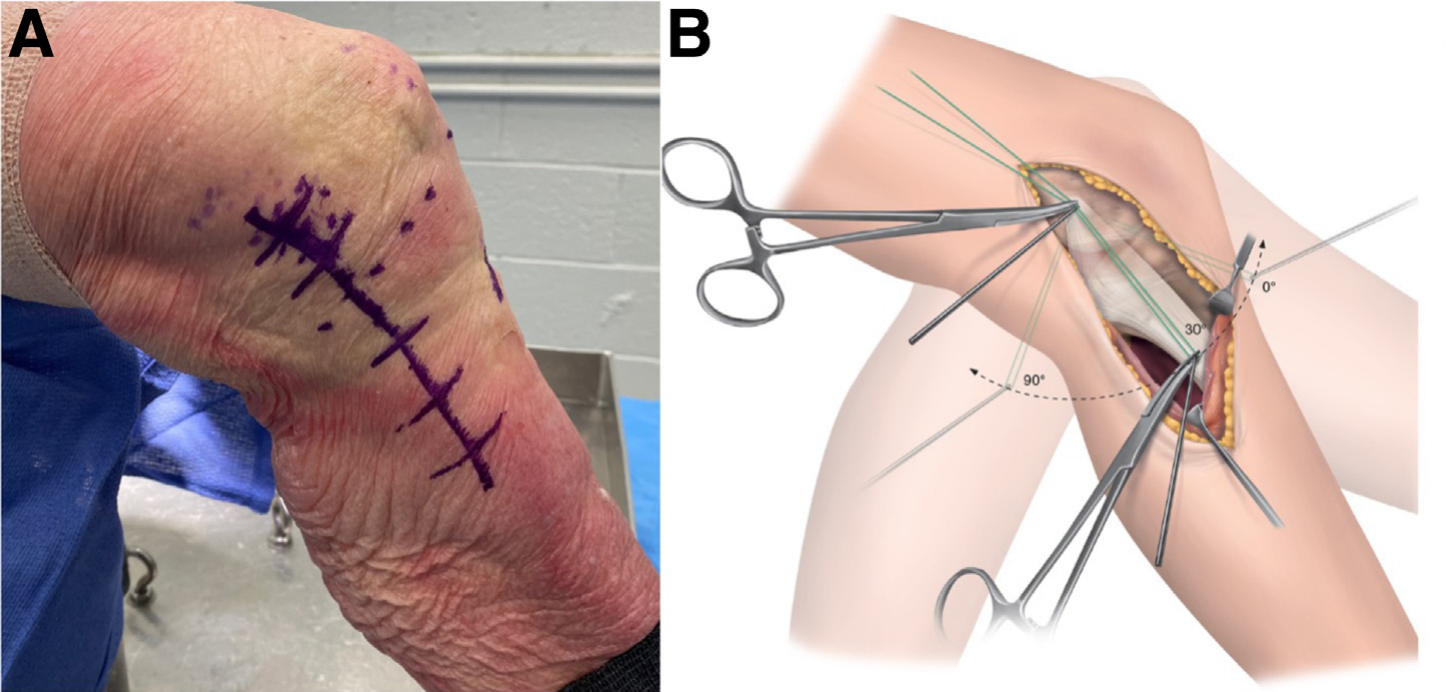
Surgical exposure and isometric testing. (A) The medial aspect of the left knee is visualized. The patient is in the supine position with the ability to both flex and extend the knee. The lower extremity is prepared and draped according to standard sterile routine. A skin incision is made extending from the medial femoral condyle to the pes anserinus (approximately 6-7 cm distal to the joint line). (B) The pes anserinus fascia is opened, and the medial collateral ligament (MCL) is visualized. A 2.0-mm K-wire is placed in the femoral origin and distal tibial insertion of the MCL. Graft isometry is checked throughout joint range of motion by wrapping a suture around both K-wires. The ends of the suture are then clamped at the proximal femoral K-wire. Suture displacement should not exceed 1 to 2 mm as the knee is ranged from flexion to extension, starting from 30°.

A skin incision is made extending from the medial femoral condyle to the pes anserinus (about 6-7 cm distal to the joint line) (Fig 1A) (Video 1). If a minimally invasive approach is preferred, 2 approximately 3-cm-long incisions will be made over the medial femoral condyle and pes anserinus.

The fascia overlying the pes anserinus is opened in such a manner that it can be closed afterward. The anterior and posterior borders of the distal MCL are visualized, and a K-wire is placed right at the center of the distal insertion of the sMCL, approximately 6 cm below the joint line and just above the pes anserinus insertion. One should note that special care must be taken to place the K-wire at the center of the distal MCL insertion because placement too far anterior or posterior will significantly affect graft isometry.

The femoral MCL insertion is visualized and palpated, and a K-wire is placed in the anatomic center of the MCL origin. The landmarks on the medial side of the femoral condyle should be studied extensively in advance as the adductor tubercle often serves as the light house of the medial side. The anatomic MCL origin can be found approximately 1 cm anterior and distal to the adductor tubercle. In case of extensive scarring after femoral MCL injury, it can be challenging to identify the correct origin of the MCL, and special care must be taken to identify the correct anatomic K-wire position to provide optimal graft isometry. To avoid tunnel collision during multiligament surgery, the K-wire should be directed 30° anteriorly and 30° proximally.

Graft isometry is checked throughout joint range of motion by wrapping a suture around both K-wires. The ends of the suture are then clamped at the proximal femoral K-wire. Suture displacement should not exceed 1 to 2 mm as the knee is ranged from flexion to extension, starting from 30° (Fig 1B).

**Fig 2:**
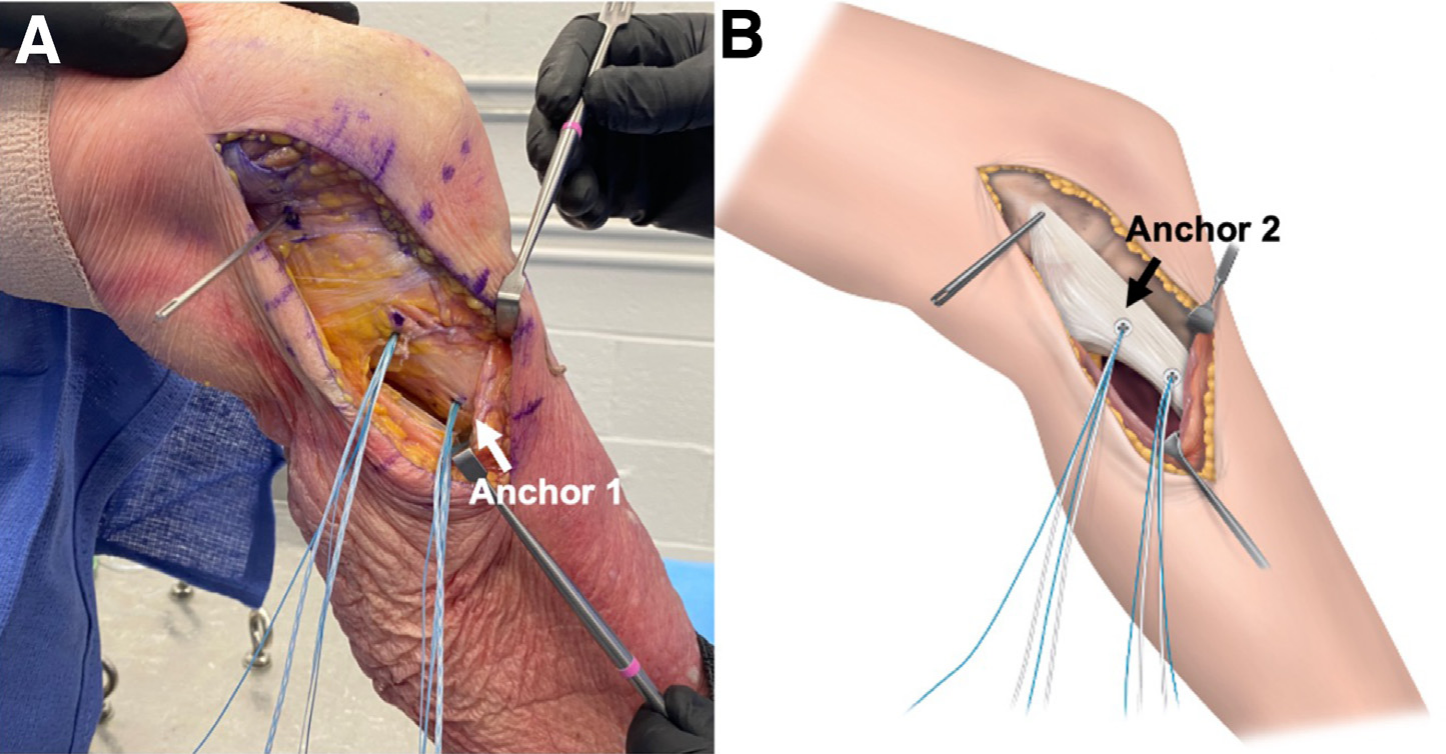
Tibial anchor insertion. (A) The medial aspect of the left knee is visualized. Once the isometric point is established, a 5.5-mm Healix Advance PEEK anchor (anchor 1) with 4 nonabsorbable sutures and needles is placed at the distal tibial insertion of the superficial medial collateral ligament (sMCL). (B) The proximal tibial insertion of the sMCL is marked using a marking pen, along with the isometric suture, followed by placement of a 5.5-mm Healix Advance BR anchor (anchor 2) with 4 nonabsorbable sutures and needles. The anchor is placed about 10 mm below the joint line, central to the course of the native MCL, at the proximal insertion of the sMCL.

Once graft isometry has been confirmed, a 5.5-mm Healix Advance PEEK (polyether ether ketone) anchor (DePuy Synthes Mitek) (anchor 1) with 4 nonabsorbable sutures and needles is placed at the distal insertion of the sMCL (Fig 2A). The proximal insertion of the sMCL is marked using a marking pen, along with the isometric suture, followed by placement of a 5.5-mm Healix Advance BR anchor (DePuy Synthes Mitek) (anchor 2) with 4 nonabsorbable sutures and needles. The anchor is placed about 10 mm below the joint line, central to the course of the native MCL, at the proximal insertion of the sMCL (Fig 2B). Thereafter, the femoral guidewire is removed and changed to a Beath pin to pass the sutures. To facilitate MCL graft and interference screw placement, care should be taken to remove all soft tissue around the bone tunnel.

### Graft Insertion and Fixation

**Fig 3:**
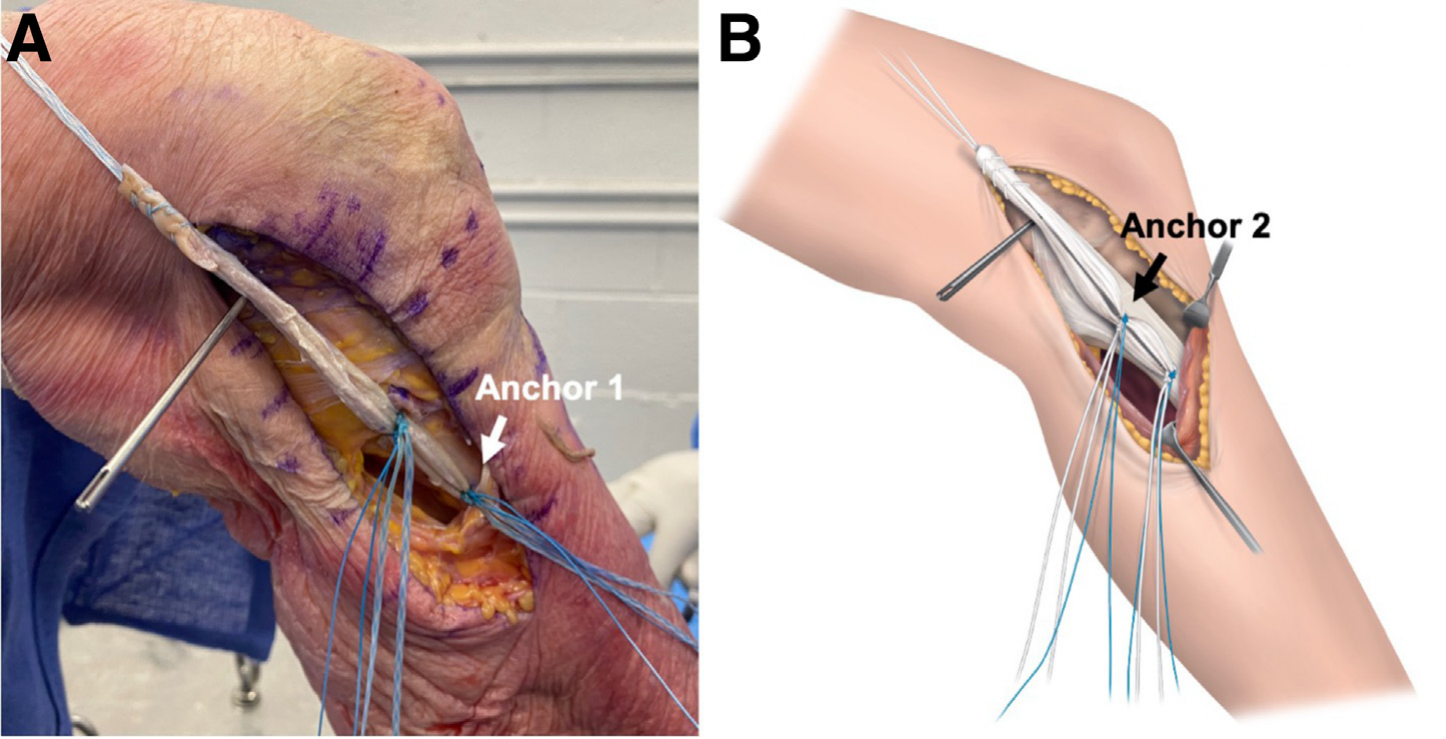
Introduction and tibial fixation of medial collateral ligament (MCL) reconstruction. (A) The medial aspect of the left knee is visualized. The allograft is placed flat along the course of the native MCL. The looped end is tied to the distal tibial superficial MCL suture anchor (anchor 1). (B) Each graft arm is additionally tied to the proximal tibial suture anchor (anchor 2) to provide a second tibial attachment closer to the joint line.

The looped MCL graft is now placed and tied firmly to the distal tibial suture anchor (anchor 1) (Fig 3). Each graft arm is additionally tied to the proximal tibial suture anchor (anchor 2) to provide a second tibial attachment closer to the joint line.

Both proximal graft ends are now whipstitched together at a length of approximately 25 to 30 mm using a No. 2 nonabsorbable suture. Any excess graft should be removed. It is recommended to insert the graft into the femoral tunnel at least 25 mm.

The femoral guidewire is then over-reamed using a drill based on the diameter of the whipstitched graft. To facilitate MCL graft and interference screw placement, care should be taken to remove all soft tissue around the bone tunnel. The surgeon should make sure to over-drill at least 10 mm extra in the tunnel so that the graft can be tensioned correctly without reaching bone.

The lead sutures are shuttled through the bone tunnel and lateral soft tissue using the Beath pin. Subsequently, the graft is inserted into the bone tunnel.

**Fig 4:**
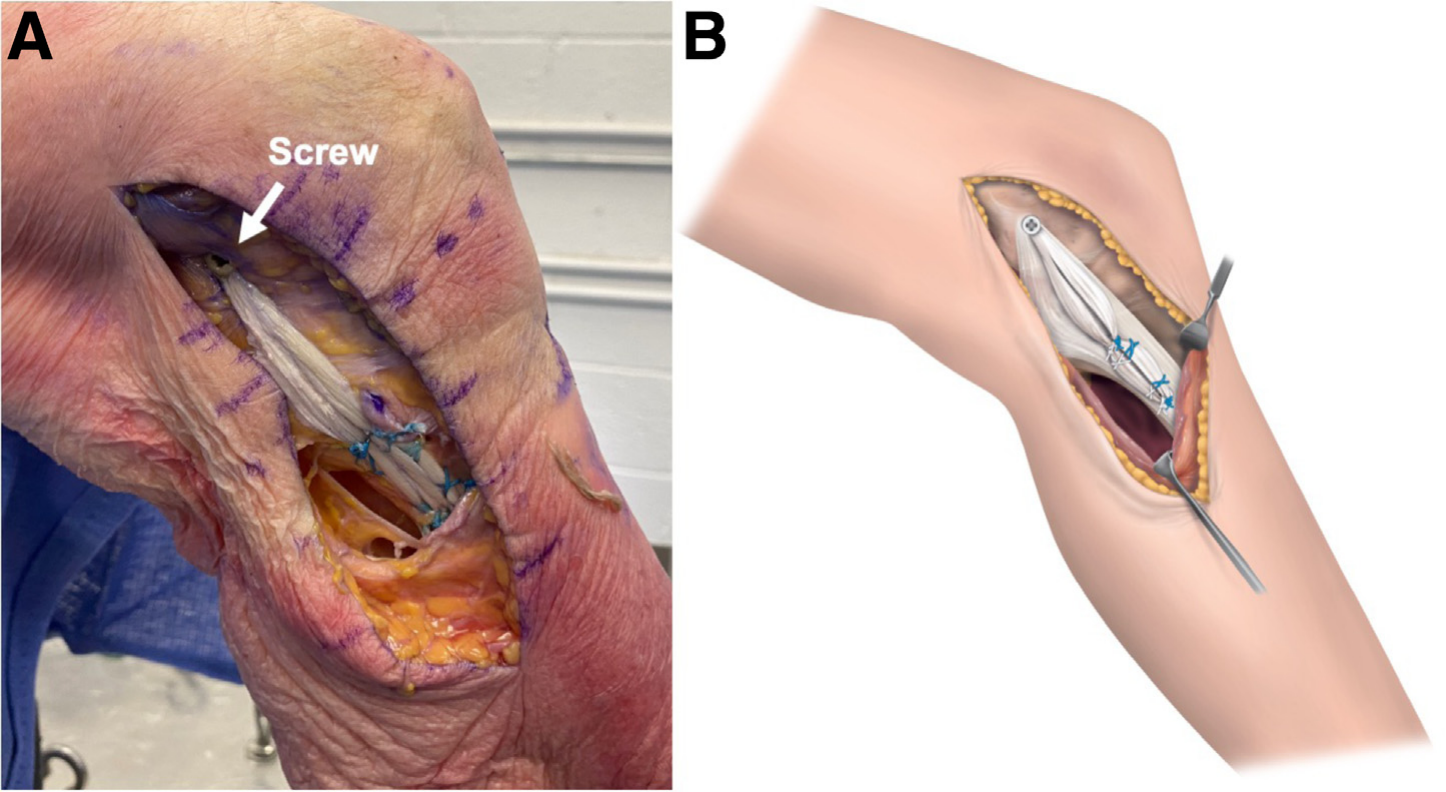
Final fixation of medial collateral ligament (MCL) allograft. (A) The medial aspect of the left knee is visualized. With the knee in 30° of knee flexion and neutral rotation, a fully threaded cannulated Bioresorbable Matryx Interference Screw is inserted over a nitinol guidewire. (B) The sutures of the proximal and distal tibial suture anchors are used to tie the MCL graft with whipstitches to the native MCL. The remaining sutures are cut to obtain the final flat double-bundle MCL reconstruction.

With the knee in 30° of knee flexion and neutral rotation, a fully threaded cannulated Bioresorbable Matryx Interference Screw (ConMed) is inserted over a nitinol guidewire (Fig 4). It should be noted that in patients with low bone mineral density or those with concerns about screw pullout (women aged > 55 years and men aged > 60 years), an additional secondary femoral fixation is advised. Our preferred method is hybrid fixation using an interference screw and an adjustable-loop cortical button; however, when an adjustable loop is used, care should be taken not to over-tension the graft.

The sutures of the proximal and distal tibial suture anchors are now used to tie the MCL graft with whipstitches to the native MCL. This provides additional fixation strength, stiffness, and flattening of the graft. The remaining sutures are cut to obtain the final flat double-bundle MCLR.

### Postoperative Rehabilitation

After operative treatment of the MCL, the patient is recommended to wear a brace for approximately 6 weeks. Range of motion and weight bearing should be limited during the first 6 weeks, with a gradual increase in knee flexion after the first week. Full weight bearing can be started as soon as it is tolerated by the patient. Starting physical therapy as early as possible after MCLR is recommended to ensure both good postoperative strength and range of motion.

## Discussion

The anatomic flat double-bundle MCLR described in this article is a surgical procedure aiming to restore medial knee stability in patients with MCL-deficient knees. Anatomic MCLR can be achieved by identifying the important landmarks for the MCL, including the femoral insertion of the MCL, as well as the tibial insertions of the sMCL (Table 1). Intraoperative isometric testing further guides the surgeon to identify the most appropriate femoral location for allograft placement and thereby ensures the isometry of MCLR without elongation and potential graft rupture over time.

**Table 1:** Pearls and Pitfalls of Anatomic Flat Double-Bundle MCLR

Pearls	Pitfalls
The surgeon should fully expose the posterior border of the tibia and femoral medial epicondyle to identify anatomic attachment sites.	Isometric points are not identified.
The surgeon should fix the distal tibial sMCL portion of the allograft first, followed by proximal tibial sMCL and femoral MCL fixation, to ensure proper length.	Additional reconstruction may be necessary in case of associated POL injury.
A 2.0-mm K-wire should be used to mark the femoral insertion point and check isometry using sutures.	The proximal and distal tibial sMCL attachment sites are not re-created.
The surgeon should ensure an adequate femoral tunnel diameter and soft-tissue dissection for smooth insertion of a whipstitched double-bundle graft.	For combined ACL-MCL reconstruction, the ACL will require autograft harvest from a different location (BPTB or quadriceps tendon autograft) if MCLR is performed with semitendinosus autograft.
The femoral interference screw should be placed while tensioned at 30° of flexion with neutral rotation and varus force.	

ACL, anterior cruciate ligament; BPTB, bone–patellar tendon–bone; MCL, medial collateral ligament; MCLR, medial collateral ligament reconstruction; POL, posterior oblique ligament; sMCL, superficial medial collateral ligament.

Biomechanical and clinical studies have previously reported increased anteromedial and rotatory instability in MCL-deficient knees, thus increasing the risk of failure after ACLR.^4^^,^^13^^,^^14^ Additionally, an up to 17 times higher risk of failure followed by revision ACLR has been reported in patients with preoperative medial instability.^8^ Currently, there are various surgical techniques described,^15^, ^16^, ^17^, ^18^ with MCLR being reported to result in superior outcomes compared with MCL repair, including decreased medial knee instability and a lower risk of failure, followed by ACLR.^9^ Yet, despite the reported advantages of MCLR in the setting of concomitant ACLR, these patients experience worse postoperative outcomes than patients undergoing isolated ACLR.^14^^,^^19^ Studies have also reported failure and persistent rotational instability after MCLR.^2^^,^^9^^,^^20^ Hence, a growing interest in isometric MCLR has recently been developed.^21^ Previous biomechanical research has illustrated the crucial role of femoral position.^21^^,^^22^ Small changes in the femoral attachment site of the transplant have been shown to lead to significant changes in graft length.^22^ This can increase the risk of nonoptimal graft tension, stretching out of the graft, and inferior outcomes.^21^^,^^22^ Therefore, this surgical technique reconstructs the MCL with allograft while evaluating the isometry of MCLR by using 2-mm K-wires. This surgical approach aims to restore medial knee stability, thereby decreasing the risk of failure when performed either alone or in combination with ACLR.

In conclusion, anatomic flat double-bundle MCLR is a treatment option for a MCL injury and we recommend performing this procedure when surgical treatment is indicated.

## Supplementary Data

ICMJE author disclosure forms

Video 1 This video demonstrates an anatomic technique for medial collateral ligament reconstruction (MCLR) performed by using flattened double-bundle allograft such as peroneus longus or semitendinosus. Intraoperative physical examination includes valgus stress at 0 and 20 degrees. The surgery begins with the patient positioned supine on the operating room table. A skin incision is made extending from the medial femoral condyle to the pes anserinus. The fascia overlaying the pes anserinus is thereafter opened in such a manner that it can be closed afterwards. The anterior and posterior border of the distal medial collateral ligament (MCL) is visualized, and a K-wire is placed at the center of the distal tibial insertion of the superficial MCL (sMCL), approximately 6 cm below the joint line. The femoral MCL insertion is visualized and palpated, and a K-wire is placed in the anatomical center of the MCL origin. Graft isometry is checked throughout joint range of motion by wrapping a suture around both K-wires. The ends of the suture are then clamped at the proximal femoral K-wire. Suture displacement should not exceed 1-2 mm as you range the knee from flexion to extension with start from 30 degrees. Once graft isometry has been confirmed, one 5.5mm Healix Advance PEEK anchors (DePuy Sytnhesis Mitek) and one 5.5mm Healix Advance BR anchors (DePuy Synthesis Mitek) with four non-absorbable sutures with needles are placed: one at the distal tibial insertion of the sMCL and the other about 10 mm below the joint line, central to the course of the native MCL, at the proximal insertion of the sMCL. The femoral guide wire is removed and changed to a Beath Pin to pass the sutures. The looped MCL allograft is thereafter placed and tied firmly to the distal tibial sMCL suture anchors. Each graft arm is additionally tied to the proximal tibial suture anchor, to provide a second tibial attachment closer to the joint line. Both proximal graft ends are now whipstitched together at a length of at least 25 mm using a no. 2 nonabsorbable suture. Any excess graft should now be removed. The femoral guidewire is then over-reamed using a drill based on the diameter size of the whipstitched graft. The lead sutures are shuttled though the bone tunnel and lateral soft tissue using the Beath Pin allowing the insertion of the graft into the bone tunnel. With the knee in 30 degrees of knee flexion and neutral rotation a fully threaded cannulated Bioresorbable Matryx Interference Screw (ConMed) is inserted over a nitinol guidewire. The sutures of the proximal and distal tibial suture anchors are now used to tie the MCL graft with whipstitches to the native MCL followed by cutting the remaining sutures to obtain the final flat double-bundle MCLR.
